# Contralesional White Matter Alterations in Patients After Hemispherotomy

**DOI:** 10.3389/fnhum.2020.00262

**Published:** 2020-07-07

**Authors:** Jennifer Gaubatz, Conrad C. Prillwitz, Leon Ernst, Bastian David, Christian Hoppe, Elke Hattingen, Bernd Weber, Hartmut Vatter, Rainer Surges, Christian E. Elger, Theodor Rüber

**Affiliations:** ^1^Department of Epileptology, University of Bonn Medical Center, Bonn, Germany; ^2^Department of Neuroradiology, Goethe University Frankfurt, Frankfurt am Main, Germany; ^3^Institute for Experimental Epileptology and Cognition Research, University of Bonn Medical Center, Bonn, Germany; ^4^Department of Neurosurgery, University of Bonn Medical Center, Bonn, Germany; ^5^Epilepsy Center Frankfurt Rhine-Main, Department of Neurology, Goethe University Frankfurt, Frankfurt am Main, Germany; ^6^Center for Personalized Translational Epilepsy Research (CePTER), Goethe University Frankfurt, Frankfurt am Main, Germany

**Keywords:** hemispherotomy, DTI, TBSS, juvenile brain lesion, plasticity

## Abstract

Cerebral lesions may cause degeneration and neuroplastic reorganization in both the ipsi- and the contralesional hemisphere, presumably creating an imbalance of primarily inhibitory interhemispheric influences produced *via* transcallosal pathways. The two hemispheres are thought to mutually hamper neuroplastic reorganization of the other hemisphere. The results of preceding degeneration and neuroplastic reorganization of white matter may be reflected by Diffusion Tensor Imaging-derived diffusivity parameters such as fractional anisotropy (FA). In this study, we applied Diffusion Tensor Imaging (DTI) to contrast the white matter status of the contralesional hemisphere of young lesioned brains with and without contralateral influences by comparing patients after hemispherotomy to those who had not undergone neurosurgery. DTI was applied to 43 healthy controls (26 females, mean age ± SD: 25.07 ± 11.33 years) and two groups of in total 51 epilepsy patients with comparable juvenile brain lesions (32 females, mean age ± SD: 25.69 ± 12.77 years) either after hemispherotomy (30 of 51 patients) or without neurosurgery (21 of 51 patients), respectively. FA values were compared between these groups using the unbiased tract-based spatial statistics approach. A voxel-wise ANCOVA controlling for age at scan yielded significant group differences in FA. A *post hoc t*-test between hemispherotomy patients and healthy controls revealed widespread supra-threshold voxels in the contralesional hemisphere of hemispherotomy patients indicating comparatively higher FA values (*p* < 0.05, FWE-corrected). The non-surgery group, in contrast, showed extensive supra-threshold voxels indicating lower FA values in the contralesional hemisphere as compared to healthy controls (*p* < 0.05, FWE-corrected). Whereas lower FA values are suggestive of pronounced contralesional degeneration in the non-surgery group, higher FA values in the hemispherotomy group may be interpreted as a result of preceding plastic remodeling. We conclude that, whether juvenile brain lesions are associated with contralesional degeneration or reorganization partly depends on the ipsilesional hemisphere. Contralesional reorganization as observed in hemispherotomy patients was most likely enabled by the complete neurosurgical deafferentation of the ipsilesional hemisphere and, thereby, the disinhibition of the neuroplastic potential of the contralesional hemisphere. The main argument of this study is that hemispherotomy may be seen as a major plastic stimulus and as a prerequisite for contralesional neuroplastic remodeling in patients with juvenile brain lesions.

## Introduction

Cerebral lesions may cause two types of structural changes in the brain: degeneration and neuroplastic reorganization (Rossini et al., 2003; Murphy and Corbett, 2009; Dodd et al., 2017). Both degeneration and neuroplastic reorganization can occur in the ipsilesional hemisphere or the contralesional, intact hemisphere (Buetefisch, 2015; Dodd et al., 2017). The concept that neuroplastic reorganization occurs in the contralesional hemisphere is particularly intriguing, as it entails the idea that one hemisphere can compensate for the losses of the other. However, this idea gets complicated by the primarily inhibitory interhemispheric influences produced *via* transcallosal pathways (Ferbert et al., 1992; Murase et al., 2004). It has been implied that inhibitory influences from the ipsilesional hemisphere hamper successful neuronal reorganization of the contralesional hemisphere and, thereby, prevent compensatory functional reorganization (Manganotti et al., 2008; Chieffo et al., 2013). In patients after hemispherotomy, the influence of the ipsilesional hemisphere on the contralesional hemisphere is completely removed: hemispherotomy is a hemispheric disconnection procedure indicated as a neurosurgical *ultima ratio* option in the treatment of selected patients with medically therapy-refractory focal epilepsy (Schramm, 2002). It has been discussed to which degree the hemispherotomy is a stimulus for neuroplastic reorganization in addition to the underlying lesion, conceivably causing structural white matter alterations (Shimizu et al., 2002; Choi et al., 2010; Nelles et al., 2015; Küpper et al., 2016). The results of preceding degeneration and neuroplastic reorganization of white matter may be reflected by Diffusion Tensor Imaging (DTI). In DTI, the MR signal is sensitized to the water diffusion rate. Directional diffusion is described as anisotropic, while directionally unrestricted diffusion is described as isotropic. DTI datasets may be used to model the underlying diffusivity characteristics in each voxel. The most common three-dimensional model mapping of anisotropic (and isotropic) diffusion is the diffusion tensor (Basser et al., 1994a,b). The most commonly used scalar DTI measure is fractional anisotropy (FA; Werring et al., 2000; Beaulieu, 2002; Puig et al., 2017). Importantly, FA may inform investigators about the microstructural white matter status. In this study, we used DTI to evaluate contralesional white matter changes after extended unilateral early brain lesions in the absence or presence of the ipsilesional hemisphere by comparing epilepsy patients who had undergone transsylvian functional hemispherotomy (hemispherotomy group) to nonsurgical patients with similar pathologies (non-surgery group). Thereby, the influence of the hemispherotomy on the plastic reorganization of the ipsilesional hemisphere could be estimated. Also, a group of healthy controls was examined. We hypothesized, that neuroplastic reorganization as indicated by higher FA could be observed in both patient groups, but was more pronounced in the hemispherotomy group, and within this group, in patients with earlier disease onset.

## Materials and Methods

### Subjects

Fifty-one epilepsy patients (32 females, mean age ± SD: 25.69 ± 12.77 years) with early acquired brain lesions (24 left-sided) and 43 age-matched healthy controls (26 females, mean age ± SD: 25.07 ± 11.33 years) with no history of neurologic or psychiatric disorders were included in this study. For the hemispherotomy group, inclusion criteria were the following: (1) treatment at the Department of Epileptology at the University of Bonn Medical Center between 1992 and 2014; (2) transsylvian functional hemispherotomy (Schramm et al., 2001) at the Department of Neurosurgery at the University of Bonn Medical Center; (3) onset of pathology and diagnosed epilepsy before the age of 8 years; (4) damage to the corticospinal system and impaired motor function; and (5) ability to undergo MR-scanning. For the non-surgery group, the inclusion criteria were the following: (1) treatment at the Department of Epileptology at the University of Bonn Medical Center between 1992 and 2014; (2) no neurosurgical procedure in patient history; (3) onset of pathology and diagnosed epilepsy before the age of 8 years; (4) damage to the corticospinal system and impaired motor function; and (5) ability to undergo MR-scanning. Thirty of the 51 patients met the inclusion criteria of the hemispherotomy group. They underwent transsylvian functional hemispherotomy at mean age 14.23 years as a treatment for pharmacoresistant epilepsy and were examined postoperatively (19 females, 16 left-sided, mean age ± SD: 23.03 ± 9.71 years). Twenty five patients of the hemispherotomy group were also analyzed as part of a previously published study (Gaubatz et al., 2020). Twenty-one patients met the inclusion criteria of the non-surgery group (13 females, eight left-sided, mean age ± SD: 29.43 ± 15.69 years). Twenty-seven of the whole group of 51 patients suffered from perinatal strokes or intracranial hemorrhages resulting in porencephaly, 12 patients were diagnosed with Rasmussen- and other encephalitides, nine patients presented neurodevelopmental disorders such as hemimegalencephaly, Sturge–Weber syndrome, polymicrogyria, or schizencephaly, two patients showed early tumor lesions and there was one patient with stroke in early childhood. In a second step, the hemispherotomy group was further divided into two subgroups according to the onset of their pathology. A cut-off age of 6 months was chosen to distinguish between an early and a late-onset subgroup (with all porencephaly patients being part of the early-onset group). The characteristics of all patients are specified in Table 1. The study was approved by the local Institutional Review Board and all participants and/or their legal guardians gave written informed consent.

**Table 1:** Overview: subjects.

Patients (*n* = 51)					
ID	Lesion Side	Hemispherotomy	Etiology	Age at scan (range)	Age at surgery (range)
**Hemispherotomy group**					
1	R	Yes	HMEG	10–14	0–4
2	L	Yes	Encephalitis*	10–14	5–9
3	L	Yes	HMEG	25–29	5–9
4	L	Yes	Porencephaly	15–19	10–14
5	R	Yes	Porencephaly	15–19	15–19
6	L	Yes	Porencephaly	20–24	10–14
7	R	Yes	Encephalitis*	45–49	30–34
8	L	Yes	HMEG	15–19	0–4
9	L	Yes	Encephalitis*	15–19	10–14
10	L	Yes	Encephalitis*	20–24	15–19
11	L	Yes	Porencephaly	15–19	10–14
12	L	Yes	Porencephaly	20–24	15–19
13	L	Yes	Porencephaly	20–24	10–14
14	R	Yes	ICH	25–29	15–19
15	L	Yes	Porencephaly	20–24	5–9
16	L	Yes	Encephalitis*	20–24	15–19
17	R	Yes	SWS	20–24	0–4
18	L	Yes	Porencephaly	15–19	5–9
19	R	Yes	Porencephaly	20–24	10–14
20	R	Yes	Porencephaly	35–39	25–29
21	R	Yes	Encephalitis*	10–14	5–9
22	R	Yes	Porencephaly	15–19	15–19
23	R	Yes	Polymicrogyria	20–24	10–14
24	R	Yes	Encephalitis*	30–34	25–29
25	R	Yes	HMEG	15–19	0–4
26	L	Yes	Porencephaly	40–44	30–34
27	L	Yes	Encephalitis*	10–14	10–14
28	R	Yes	Encephalitis*	30–34	5–9
29	L	Yes	Porencephaly	45–49	45–49
30	R	Yes	Tumor	25–29	25–29
**mean ± SD**	16 left			23.03 ± 9.71	14.23 ± 11.01
**Non-surgery group**					
31	L	No	Porencephaly	30–34	-
32	L	No	Porencephaly	60–64	-
33	R	No	Porencephaly	10–14	-
34	R	No	Porencephaly	60–64	-
35	R	No	Encephalitis*	30–34	-
36	R	No	Stroke*	25–29	-
37	R	No	Porencephaly	45–49	-
38	L	No	Polymicrogyria	20–24	-
39	L	No	Porencephaly	10–14	-
40	L	No	Porencephaly	20–24	-
41	R	No	Encephalitis*	45–49	-
42	L	No	Porencephaly	25–29	-
43	R	No	Polymicrogyria	20–24	-
44	R	No	Encephalitis*	15–19	-
45	R	No	Porencephaly	15–19	-
46	L	No	Porencephaly	25–29	-
47	R	No	Porencephaly	45–49	-
48	R	No	Schizencephaly	20–24	-
49	R	No	Porencephaly	30–34	-
50	L	No	Porencephaly	15–19	-
51	R	No	Tumor	10–14	-
mean ± SD	8 left			29.43 ± 15.69
**All Patients**					
mean ± SD	24 left	30 yes		25.69 ± 12.77
**Healthy controls (*n* = 43)**					
mean ± SD				25.07 ± 11.33

*Abbreviations: F, female; M, male; L, left; R, right; HMEG, Hemimegalencephaly; ICH, intracranial hemorrhage; SWS, Sturge–Weber syndrome. Etiologies marked with an asterisk occurred after the age of 6 months and were considered as late-onset*.

### Image Acquisition

Magnetic resonance imaging data was acquired using a 3T Magnetom Trio (Siemens Healthineers). T1, T2 and DTI data were collected for all patients and healthy controls. As part of a scanner update, a new head coil was implemented in October 2014 leading to minimal changes in sequence parameters: all scans acquired before the scanner update were run with an eight-channel-coil and a scanning routine containing a 3D MPRAGE sequence (resolution = 1.0 × 1.0 × 1.0 mm^3^, TR = 1,570 ms, TE = 3.42 ms, flip angle = 15°), a 3D T2-weighted sequence (resolution = 1.0 × 1.0 × 1.0 mm^3^, TR = 3,200 ms, TE = 455 ms, flip angle = 120°), and a single-shot diffusion-weighted sequence (resolution = 1.72 × 1.72 × 1.7 mm^3^, TR = 12,000 ms, TE = 100 ms, flip angle = 90°) with 60 diffusion-encoding directions and a *b*-value of 1,000 s/mm^2^ as well as six baseline volumes with a *b*-value of 0 s/mm^2^. Sequences acquired after the scanner update were run with a 32-channel head-coil equally including a MPRAGE sequence (resolution = 0.8 × 0.8 × 0.8 mm^3^, TR = 1,660 ms, TE = 2.54 ms, flip angle = 9°), T2-weighted sequence (resolution = 0.8 × 0.8 × 0.8 mm^3^, TR = 3,200 ms, TE = 401 ms, flip angle = 120°), and a single-shot diffusion-weighted sequence (resolution 1.72 × 1.72 × 1.7 mm^3^, TR = 9,000 ms, TE = 87 ms, flip angle = 90°) with 60 diffusion-encoding directions and a *b-value* of 1,000 s/mm^2^ as well as six baseline volumes with a *b-value* of 0 s/mm^2^.

### Preprocessing

Preprocessing was run using FMRIB’s Software Library 5.0 (Jenkinson et al., 2012) and the Tolerable Obsessive Registration and Tensor Optimization Indolent Software Ensemble (TORTOISE; Pierpaoli et al., 2010). In a first step, T1- and T2-weighted volumes were brain extracted (Smith, 2002) and corrected for intensity inhomogeneities (Zhang et al., 2001). As extensive lesions, as present in our study group, tend to bias the normalization process, individual masks covering compromised structures of the lesional hemisphere were manually drawn on the T1-weighted volumes. By inverting those masks, only healthy tissue was considered during the computation of all inter-individual transformations. Thus, T1-weighted volumes were normalized to the Montreal Neurological Institute template (MNI152 nonlinear, 6th generation, 1 mm resolution) using linear and non-linear registration (Jenkinson and Smith, 2001). For DTI volumes, susceptibility-induced geometric distortions were corrected using constrained registration (Bhushan et al., 2012) on the same step as motion and eddy current correction. Diffusion tensors were fitted to each voxel in the corrected DTI data and FA was calculated. Finally, the mean of each patients’ six *b0* baseline volumes was calculated as a reference image for a boundary-based registration (BBR) to the respective T1-weighted volume.

### TBSS

**Figure 1:**
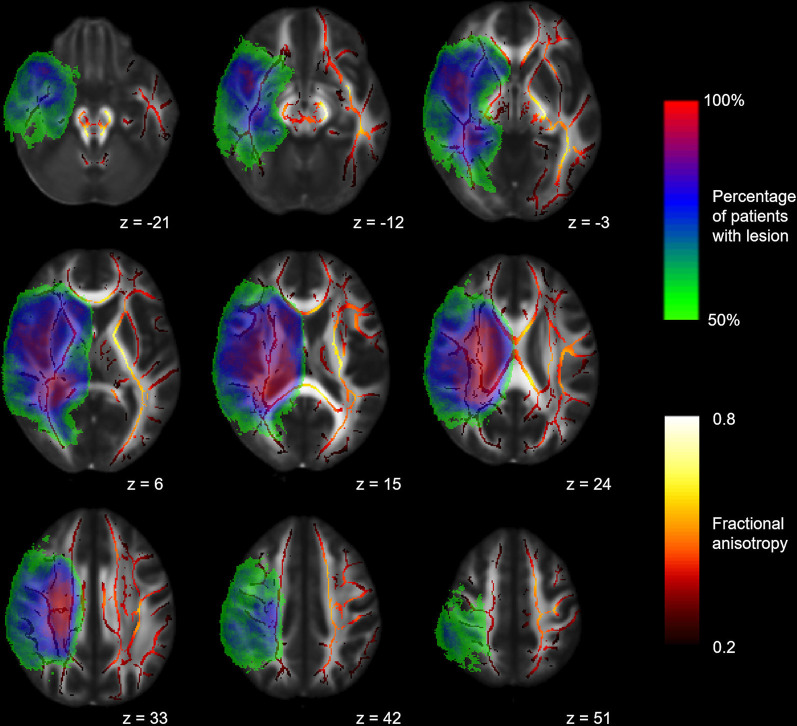
Mean fractional anisotropy (FA) skeleton and canonical lesion mask. Mean FA skeleton (red to yellow) of all subjects and canonical lesion mask (rainbow) created from all patients shown on the FMRIB58_FA template. Volumes of patients with left-hemispheric lesions are flipped along the x-axis. Coordinates are provided in MNI standard space.

Here, we opted to analyze our datasets using tract-based spatial statistics (TBSS; Smith et al., 2006). TBSS is part of the FMRIB’s Software Library 5.0 and has been developed to face two of the biggest challenges of conventional voxel-wise analysis in DTI analysis: multiple comparison and alignment. In TBSS, voxel-wise analysis is constrained to the FA skeleton, which represents the centers of all white matter tracts common to the study group (Andersson et al., 2007a,b). This FA skeleton is applied to mitigate any residual misalignment after a common mapping function has been applied. In the standard processing pipeline, individual FA maps are normalized applying a nonlinear transformation. Normalization describes the process of aligning an individual brain to a template brain using a special function. In the process of normalization, every voxel is assigned specific coordinates (Snook et al., 2007). After normalization, these coordinates are supposed to correspond to certain anatomical structures. At the end of successful normalization, all brains align well with each other. In the TBSS standard routine, a mean FA map (in standard space) is generated based on all normalized brains and used to create the white matter skeleton. Next, the skeleton is projected on the individual FA map but is deformed based on a local search for maximal FA values. Lastly, voxel-wise testing may be performed within the skeleton as described above. It is not surprising that the registration of healthy brains will never work perfectly, let alone the registration of lesioned brains. The general advice to create a study-specific template (Smith et al., 2006) would mean the following for our study: Non-linear transformation of each FA map to the “most representative” one of the group, affine-alignment to the MNI152 space and transformation of every image into 1 × 1 × 1 mm MNI152 space by combining the warp-field to the target FA image with the affine transformation from that target to MNI152 space. However, since most brains of the patient group presented extensive lesions, a “most representative” image cannot be determined. Instead, each FA-map was transformed to MNI152 space by combining the above mentioned BBR-based registration of the individual mean *b0* volume to the respective T1-weighted image with the warp-field aligning this T1-weighted image to MNI152 space. Volumes of patients with lesions in the left hemisphere were flipped along the x-axis, allowing voxel-wise comparisons within the healthy hemisphere of all patients. For between-group comparisons, the same proportions of healthy controls as in the patient groups, respectively, were flipped along the x-axis to exclude the effects of physiological brain asymmetry. Lesion masks were delineated manually on the individual T1 volumes in native space and controlled by a second, independent rater. Normalized lesion masks were binarized and merged to create a canonical lesion mask. This canonical lesion mask was modified with a 50% threshold and used to restrain the TBSS skeleton in the lesional hemisphere to exclude deformed tissue structures from the analysis (see Figure 1).

### Statistics

Statistical analyses were performed with FSL’s tool for nonparametric permutation inference (Winkler et al., 2014). To test whether the FA values of the hemispherotomy group, the non-surgery group, and the healthy control group would show significant differences, we ran a one-factor, three-level analysis of covariance (ANCOVA). *Age at scan* and a variable indicating whether subjects had been scanned before or after the scanner update were both included as covariates. Directions of effects shown in the overall *F-test* were determined with three *post hoc* unpaired (two-sample) *t*-tests between all groups. Group effects in all *post hoc t*-tests were equally adjusted for *scanner update* and *age at scan*. The anatomical location of resulting clusters was identified with the JHU ICBM-DTI-81 white-matter labels atlas (Mori et al., 2005). In a second approach, FA values were subjected to a two-sample unpaired *t*-test between subgroups of hemispherotomy patients with an early (21 patients, 13 females, mean age ± SD: 23.29 ± 9.30 years) and late (nine patients, five females, mean age ± SD: 25.11 ± 11.11 years) onset of pathology. *Scanner* *update* and *age at surgery* were distributed equally in both subgroups and, thus, had not to be adjusted for this model. Lastly, we performed a voxel-wise regression analysis to link the volume of the lesion masks to voxel-wise FA values. All analyses were run using Monte Carlo permutation tests with 10,000 permutations. As we performed voxel-wise testing, as many statistical tests as there are voxels in the white matter skeleton were carried out. To keep the conventional level of significance at 5% and to control for the number of false positives, results were corrected for family-wise error if not indicated otherwise. Threshold-free cluster enhancement was performed to avoid an arbitrary initial cluster-forming threshold for final voxel-wise inference (Smith and Nichols, 2009). Resulting clusters were thickened to aid visualization.

## Results

### Intergroup FA Differences

**Figure 2:**
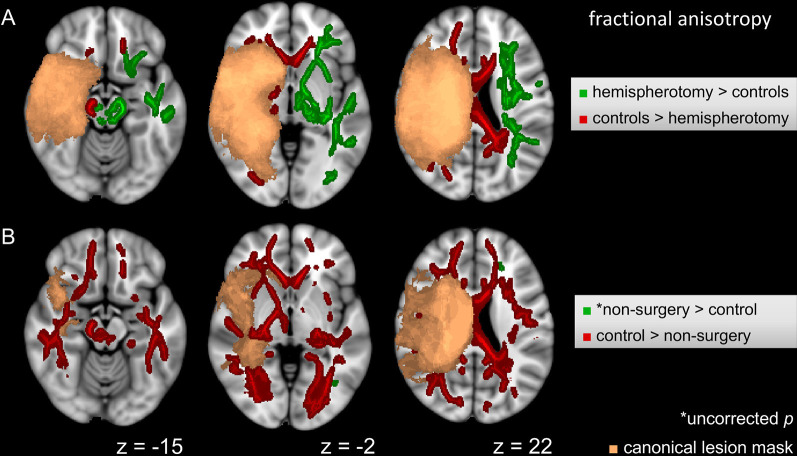
Intergroup FA differences. **(A)** Voxel-wise post hoc tests in FA between hemispherotomy group and healthy controls. **(B)** Voxel-wise *post hoc* tests in FA between non-surgery group and healthy controls. If not indicated otherwise, tests were corrected for family-wise error and *p* < 0.05. z indicates axial coordinate in MNI standard space. Please note that the canonical lesions masks differ between the post hoc tests as they are built out of the individual lesion masks of the respective groups contrasted. Skeletonized results were thickened for visualization purposes.

The voxel-wise ANCOVA controlling for *scanner* *update* and *age at scan* as covariates yielded significant effects of the group factor (*p* < 0.05): clusters were widely distributed in the contralesional hemisphere, whereas they only covered frontal areas and cerebral peduncle in the lesional hemisphere. Adjusting for *scanner update* and *age at scan*, FA values were subjected to two *post hoc t*-tests comparing the hemispherotomy group and the healthy control group (A) and the non-surgery group and the healthy control group (B). A: widespread clusters indicating higher FA values in hemispherotomized patients as compared to healthy controls were found primarily in the internal capsule, the external capsule, superior and posterior corona radiata, posterior thalamic radiations, and brainstem regions in the contralesional hemisphere (*p* < 0.05; see Figure 2A, green). In the ipsilesional hemisphere, clusters indicating comparatively higher FA values in the hemispherotomy group were only found in small parts of the brainstem (which had not been deafferented). Comparatively lower FA values in the hemispherotomy group were found in residual callosal pathways, in perilesional frontal and occipital regions as well as in the ipsilesional medullary pyramid (see Figure 2A, red). B: clusters indicating lower FA values in the non-surgery group as compared to healthy controls resembled those confirming lower FA values of the hemispherotomy group as compared to healthy controls in A (Figure 2B, red). In B however, clusters were more widespread as they covered larger parts of the temporal and occipital lobes. Only very small clusters proving higher FA values in non-surgery patients as compared to healthy controls emerged in the contralesional hemisphere and did not survive correction for multiple comparisons (Figure 2B, green), whereas results of all other contrasts applied to show intergroup FA differences were corrected for family-wise error.

### Intragroup FA Differences Within the Hemispherotomy Group

**Figure 3:**
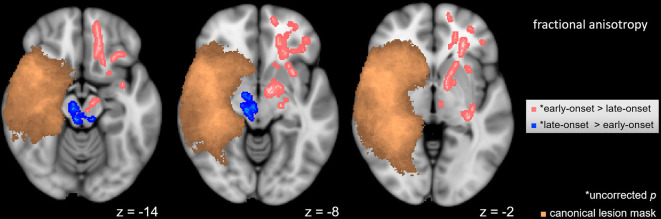
Intragroup FA differences. Voxel-wise *t*-test in FA between hemispherotomy subgroups with early-onset and late-onset pathologies (*p* < 0.05, uncorrected for multiple comparisons). Copper voxels indicate a canonical lesion mask. z indicates axial coordinate in MNI space. Skeletonized results were thickened for visualization purposes.

A voxel-wise *t*-test (*p* < 0.05) was used to determine differences between subgroups of hemispherotomy patients with early and late-onset of pathology. Patients with late-onset of pathology (>0.5 years old at onset) exhibited higher FA values in residual ipsilesional brainstem regions (Figure 3, blue), while patients with early onset of pathology (<0.5 years old at onset) presented higher FA values in frontal regions and within the corona radiata of the contralesional hemisphere (Figure 3, pink). Results of the intragroup comparison did not survive correction for multiple comparisons. The opposite contrasts did not yield significant results.

### Association Between FA Alterations and Lesion Size

A voxel-wise regression analysis aiming to explain FA values by lesion size did not yield statistically significant results (*p* > 0.45), even when omitting a correction for multiple comparisons (*p* > 0.10).

## Discussion

In this study, we contrast contralesional FA differences of two epilepsy patient groups with juvenile brain lesions after hemispherotomy and without neurosurgery. Our hypothesis was partly confirmed by the comparison between patients after hemispherotomy and healthy controls resulting in widespread supra-threshold voxels which indicated higher FA values and most likely extensive neuroplastic reorganization in the hemispherotomy group. However, the same comparison with the non-surgery group left us with widespread supra-threshold voxels indicating comparatively lower FA values, most likely indicating more pronounced degeneration in nonsurgical patients, which is not what we hypothesized. Hemispherotomy/the removal of ipsilesional influences seems to be a prerequisite for contralesional neuroplastic remodeling.

### Contralesional White Matter Alterations With and Without Ipsilesional Influences

These results alone do not allow for definite inferences to be drawn, however, they yield insights when put in the context of previous studies. It is a common notion that changes in white matter microstructure happen within a genetically determined framework (Rüber et al., 2016). This framework changes across the lifespan leaving varying room for the white matter effects of the environment. DTI may detect even subtle white matter alterations, however, it remains challenging to specifically attribute these alterations to the influence of genetic factors, environment, or age (Rüber et al., 2016). It is a common notion that illness, aging and a loss of cognitive functions is associated with decreased FA values, whereas learning and rehabilitation are accompanied by increased FA values (Bengtsson et al., 2005; Landi et al., 2011; Engvig et al., 2012; Lebel et al., 2012; Zheng and Schlaug, 2015). Previous studies have been quite controversial concerning the role of the contralesional hemisphere in functional recovery after extended unilateral brain lesions. It is easy to conceive that the contralesional hemisphere is the only possible compensator left in patients after hemispherotomy. In patients with unilateral brain lesions but without deafferentation, some imaging studies have found no signs of structural white matter affection of the contralesional hemisphere (Lindenberg et al., 2010a; Rüber et al., 2012), whereas others found DTI indices of contralesional structural reorganization partly correlating with the degree of motor recovery (Schaechter et al., 2009; Crofts et al., 2011). It should be noted that beside contralesional reorganization, also contralesional degeneration has been observed, most likely constituting the structural correlate of what has been termed diaschisis (von Monakow, 1914; Seitz et al., 1999): the post-lesional change of function constituted in brain areas remote from the anatomical site of damage.

**Figure 4:**
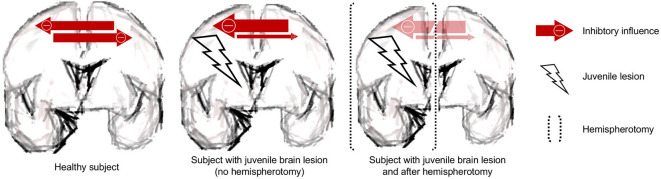
Schematic. The juvenile brain lesion creates an interhemispheric imbalance with prevailing inhibitory influences. These inhibitory influences are removed by the procedure of hemispherotomy.

Apart from our results, much speaks in favor of the underlying lesion being a powerful stimulus for neuroplastic reorganization: most convincingly, language organization is induced in the right hemisphere when the lesion has occurred to the left one (Staudt et al., 2002b). Furthermore, a large group of hemispherotomy patients shows unchanged motor scores directly after surgery (Choi et al., 2010; Küpper et al., 2016), while some groups reported even better postoperative motor skills after hemispherotomy (Shimizu et al., 2002; van der Kolk et al., 2013; Küpper et al., 2016). Also, patients with pre-operative mutism have anecdotally been reported to acquire speech after hemispherotomy (Vargha-Khadem et al., 1997; Bulteau et al., 2017; two non-published cases in our department). It can easily be seen how the neuroplastic reorganization of the contralesional hemisphere is hampered by the transcallosal inhibition. In an attempt to diminish interhemispheric imbalance, the ipsilesional hemisphere conceivably shows abnormal hyperexcitability. A recent transcranial magnetic stimulation (TMS) study suggests that the recovery of stroke patients is achieved by the excitability increase of the contralesional hemisphere (Stinear et al., 2015). Higher excitability of the ipsilesional hemisphere equals stronger inhibition of the contralesional hemisphere. The surgical deafferentation would, thus, result in a disinhibition of the contralesional hemisphere allowing the contralesional hemisphere to undergo substantial neuroplastic reorganization (Shimizu et al., 2002). The hemispherotomy in that sense may allow unmasking of the plastic potential of the contralesional hemisphere by taking full advantage of what has been termed “degeneracy”—the ability of structurally nonidentical biological elements (like the two hemispheres) to exert the same function (Noppeney et al., 2004). See Figure 4 for a schematic.

Degeneracy is a biological principle of paramount importance for post-lesional recovery. Additionally, neurorehabilitation may leverage the understanding of interhemispheric influences in the lesioned brain. A burgeoning body of evidence (Hummel and Cohen, 2006; Lindenberg et al., 2010b; Grefkes and Fink, 2016; Ladenbauer et al., 2017) from studies suggests that non-invasive brain stimulation such as transcranial direct current stimulation (tDCS) or TMS may enhance recovery. By downregulating the excitability of the contralesional hemisphere, by upregulating the excitability of the ipsilesional hemisphere or by combining both recoveries after stroke may be facilitated. Non-invasive brain stimulation has not been applied to hemispherotomy patients, however, our results inspire trials aimed at “preparing” patients for surgery by downregulating the ipsilesional hemisphere and anticipating the postoperative status.

### Early vs. Late Onset of Pathology

The obtained effect of age at onset on FA values is in line with the previously mentioned idea that early brain lesions may lead to the reinforcement of uncrossed pyramidal tract of the ipsilesional hemisphere: the pyramidal tract is bilaterally organized at birth, but uncrossed connections are normally pruned around the age of one and a half years (Eyre et al., 2001). In patients with early lesions, these normally transient unilateral connections may be preserved (Staudt et al., 2002a) and the contralesional hemisphere overtakes motor function. The preservation of these connections is most likely reflected by comparatively higher FA values in the contralesional frontal lobe, whereas patients with late lesions had to recruit ipsilesional regions for functional compensation as indicated by comparatively higher FA values in the residual ipsilesional brainstem. As it is too late for the preservation of the (contralesional) uncrossed pyramidal tract, the ipsilesional hemisphere itself has to compensate the lesion.

### Limitations

However, one should be wary when interpreting our results concerning the fact that some variables known to influence the white matter status could not be controlled for: patients after hemispherotomy may not necessarily have larger lesions, however, it may be guessed that they have a higher seizure burden (which ultimately lead to the indication for hemispherotomy) and a higher antiepileptic drug load, both thought to lower FA values (Jehi, 2017). In the hemispherotomy group, however, comparatively higher FA values have been found. Another constraint of this study is set by the restrictive limits of DTI in the assessment of white matter microstructure. On a microstructural scale, the relation between DTI indices and their biological substrates remains to be elucidated. It is important to acknowledge that there is no “one-to-one association” between any DTI index and a single white matter component (Johansen-Berg and Rushworth, 2009). Distinct microstructural processes may lead to the same DTI observations since there is a coexistence of different biological substrates influencing each other (Cercignani and Gandini Wheeler-Kingshott, 2018). The findings of this study shall, thus, not directly be interpreted as correlates of distinct microstructural processes, but rather as indirect correlates of loss of function or recovery previously defined by previous studies. It is a limitation of this study, that no function-structure associations were shown. Furthermore, results of the subgroup-comparison were not corrected for multiple comparisons and we cannot overemphasize the fact that they should, thus, be interpreted with great caution. Lastly, the most important limitation of our study lies in the heterogenicity of the study groups. The number of patients with lesions in the left and the right hemisphere was not balanced and also there were several different etiologies present. Regarding the unbalanced lesion sides in the patient group, it should be noted that in this study, patient brains and an equal proportion of the respective control group were flipped along the x-axis as part of the preprocessing. While this step is inevitable for a joint analysis of the contralesional hemisphere, it has been shown that the white matter architecture of the brain shows a left > right asymmetry in FA (Takao et al., 2011). In a DTI twin study (Jahanshad et al., 2010), 20% to 40% of FA brain asymmetry was even found to be genetically determined. The asymmetry of white matter architecture is being accounted for by flipping the equal proportion of the respective control group of healthy subjects, however, what we do not know is whether a left hemisphere changes differently after lesion/hemispherotomy occurs to the right hemisphere as compared to a right hemisphere after lesion/hemispherotomy occurs to the left one. Regarding the different pathologies in the patient groups (perinatal strokes or intracranial hemorrhages resulting in porencephaly = 27 patients, Rasmussen- and other encephalitides = 12 patients, hemimegalencephaly, Sturge–Weber syndrome, polymicrogyria, or schizencephaly = 9 patients, early tumor lesions = 2 patients), it is likely that they will have different effects on white matter degeneration and reorganization. Whereas the lesion size does not seem to be influential on white matter diffusivity characteristics (as indicated by our regression analysis, see “Association Between FA Alterations and Lesion Size” section), the time onset of the lesion along with the lifespan surely is (as seen in the comparison between patients with early and late-onset pathology). While the concept of an all-decisive “critical phase” in early childhood with a later loss of plastic potential has been softened, partly due to imaging studies with older patients (Jäncke, 2009), the core of the so-called Kennard principle (Kennard, 1936) with a higher plastic potential in early childhood or adolescence still applies (Dennis, 2010). However, one has to keep in mind that hemispherotomy is a neurosurgical procedure extremely rarely performed. Between 100 and 160 yearly hemispherotomies/hemispherectomies have been estimated to be performed in the United States between 2000 and 2009 (Lin et al., 2015). It is, thus, extremely difficult to ascertain a homogenous group of hemispherotomy patients large enough to perform analyses with sufficient statistical power. It is also for this reason that we opted to use TBSS for the analysis of our datasets, as TBSS gains gain statistical power from the dimensionality reduction of projecting FA values on the white matter skeleton (Bach et al., 2014). While this fact explains our limitations, it does not overcome them. A future longitudinal study investigating how white matter microstructure changes before and after hemispherotomy would be one way to overcome some of the limitations of the current study.

## Conclusion

Lower FA values unexpectedly found in the white matter infrastructure of the contralesional hemisphere of epilepsy patients with juvenile brain lesion when compared to healthy controls most likely reflect secondary degeneration and may constitute the structural correlate of diaschisis. This degeneration may be aggravated and neuroplastic reorganization in this hemisphere may be hindered by the inhibitory influence of the ipsilesional hemisphere produced *via* transcallosal pathways. The contrasting result of higher FA values in the contralesional hemisphere in a group of patients with similar pathologies who had undergone hemispherotomy as compared to healthy controls may be interpreted as a correlate of preceding neuroplastic reorganization of the contralesional hemisphere. This reorganization has most likely been enabled by the lacking inhibitory influence of the ipsilesional hemisphere resulting in the disinhibition of the contralesional one. Our study probes models of interhemispheric balance by applying it to two patient groups with early brain lesions after and without hemispherotomy. The pattern of degenerative and plastic structural alterations is most likely a result of interhemispheric inhibition and, therefore, substantiates these models. We conclude, that the procedure of hemispherotomy should be seen as a major plastic stimulus for reorganization in the contralesional hemisphere. Complete disconnection of inhibitory influences from the ipsilesional hemisphere releases the full rehabilitative potential of the contralesional hemisphere for functional recovery in patients with extended unilateral lesions.

## Data Availability Statement

The data that support the findings of this study are available on request from the corresponding author. The data are not publicly available as they contain information that could compromise the privacy of research participants.

## Ethics Statement

The studies involving human participants were reviewed and approved by Ethics Committee of the Medical Faculty of the University of Bonn. Written informed consent to participate in this study was provided by the participants’ legal guardian/next of kin.

## Author Contributions

JG, CP, EH, BW, HV, RS, CE, and TR contributed to the conception and design of the study. JG, CP, and LE organized the database. JG, LE, BD, and TR performed the statistical analysis. JG wrote the first draft of the manuscript. CH and TR wrote sections of the manuscript. EH, BW, HV, RS, CE, and TR gave administrative, technical or material support. EH, BW, HV, RS, CE, and TR supervised the study. All authors contributed to manuscript revision, read and approved the submitted version. JG and CP have contributed equally to this work.

## Conflict of Interest

The authors declare that the research was conducted in the absence of any commercial or financial relationships that could be construed as a potential conflict of interest.
